# Accuracy of predictive equations for resting energy expenditure estimation in mechanically ventilated Thai patients

**DOI:** 10.2478/abm-2023-0041

**Published:** 2023-08-07

**Authors:** Napplika Kongpolprom

**Affiliations:** Division of Pulmonary and Critical Care Medicine, Department of Medicine, Faculty of Medicine, Chulalongkorn University, Bangkok 10330, Thailand

**Keywords:** calorimetry, indirect, energy metabolism, expenditures, energy, ventilation, critical illness

## Abstract

**Background:**

Indirect calorimetry (IC) is the most precise approach for estimating calorie demand in critically ill patients. Despite this, owing to unaffordable devices, it is rarely used in practice. Predictive equations are the alternatives.

**Objectives:**

To assess the accuracy of 14 predictive resting energy expenditure(REE) equations in ventilated Thai patients.

**Methods:**

We compared the accuracy and agreement of 14 equations. The equations included the American College of Chest Physicians(ACCP) equation, Harris–Benedict equation(HBE), 1.2×HBE, 1.5×HBE, Mifflin–St. Jeor(MSJ), Ireton-Jones 1992 and 2002, Penn State 2003(HBE and MSJ) and 2010, Swinamer 1990, Faisy, Brandi 1999, and 25 kcal/kg equation. An equation was ascertained as accurate if the calculated values fell within ±10% of the measured REEs. Spearman correlation coefficient, Bland–Altman method, and intraclass correlation coefficient were used to analysis.

**Results:**

We obtained data from 24 ventilated patients undergoing REE measurement by IC. Fifty percent of them were male with a median age of 64.5 years, a median height of 160 cm, and a median body mass index of 22.95 kg/m^2^. The predictive precision of all equations was poor, with largely different accuracies from 6.7% to 48.1%. The most reliable equation was Penn State 2010. The ACCP, HBE, MSJ, and Penn State 2003(HBE) tended to underestimate calorie need. Contrastingly, the other equations tended to overestimate REEs. Despite a moderate degree of correlations, the Bland–Altman plots demonstrated clinically unacceptable discrepancies between measured REE and REE calculated by each equation.

**Conclusions:**

In ventilated Thai patients, there were no precise equations for determining REE.

Indirect calorimetry (IC) is the gold standard for determining calorie need in critically ill patients [1, 2, 3]. However, it is rarely affordable in Thailand. In routine clinical practice, predictive equations are the alternatives. The precision of previously validated equations in the estimation of energy requirements in critically ill patients varied from 37% to 65% of measured resting energy expenditure (REE) [4]. Among the various equations available for use in determining the calorie demand and thus achieving nutritional support optimization, Thai physicians commonly use the American College of Chest Physicians (ACCP) equation [5]. However, the use of any of these equations might result in inaccurate estimation of REE in Thai patients. To our knowledge, there has been no equation validated for mechanically ventilated Thai patients.

We aimed to evaluate the accuracy and agreement of 14 predictive equations for estimating REE with measured IC in mechanically ventilated Thai patients.

## Materials and methods

### Study design

We conducted a retrospective study of the accuracy of predictive REE in mechanically ventilated Thai patients. We reviewed data, including measured REEs and other variables used for REE calculation, pertaining to ventilated patients admitted in medical intensive care units in King Chulalongkorn Memorial Hospital between June 2014 and May 2016. We determined the accuracy and agreement of 14 predictive equations, as indicated in **Table 1** [4, 5, 6, 7, 8, 9, 10, 11, 12, 13]. The clinical variables used for the calculations involved in the equations, such as heart rate (HR), minute ventilation, body temperature (BT), etc., were derived from the same REE measurement period. This study was approved by the Institutional Review Board (IRB) of the Faculty of Medicine, Chulalongkorn University, for authorization for medical record review (certificate of approval no. 493/60). The clinical trial registration number was TCTR20171014004.

**Table 1.** Predictive equations for REE

**Equations**	**Formula**
ACCP equation	= 25 × weight If BMI is in the range of 16–25 kg/m^2^, use usual BW If BMI > 25 kg/m^2^, use IBW If BMI < 16 kg/m^2^, use existing BW for the first 7–10 d, and then use IBW
HBE	Men: kcal/d = 66.47 + 13.75 (w) + 5 (h) − 6.75 (a) Women: kcal/d = 655.1 + 9.56 (w) + 1.85 (h) − 4.68 (a) w = actual weight (kg); but if >125% IBW, use ABW ABW = [(actual body weight − IBW) × (0.25 to 0.5)] + IBW
MSJ equation	Men: kcal/d = 10 (w) + 6.25 (h) − 5 (a) + 5 Women: kcal/d = 10 (w) + 6.25 (h) − 5 (a) − 161
Ireton-Jones 1992 equation	1925 − 10 (a) + 5 (w) + 281 (S) + 292 (T) + 851 (B)
Ireton-Jones 2002 equation	1784 − 11 (a) + 5 (w) + 244 (S) + 239 (T) + 804 (B)
Penn State 2003 equation	Harris–Benedict: kcal/d = 0.85 (HBE) + 175 (Tmax) + 33 (Ve) − 6433 Mifflin: kcal/d = 0.96 (MSJ) + 167 (Tmax) + 31 (Ve) − 6212
Penn State 2010 equation	MSJ (0.71) + Ve (64) + Tmax (85) − 3085
Swinamer 1990 equation	(945 × BSA) − (6.4 × age) + (108 × BT) + (24.2 × RR) + (817 × VT) − 4349
Faisy equation	8 (w) + 14 (h) + 42 (Ve) + 94 (BT) − 4834
Brandi 1999 equation	0.96 (HBE) + 7 (HR) + 48 (Ve) − 702
Equation commonly used in critical illness	25 kcal/kg actual body weight including 1–1.5 g protein/kg

The clinical variables such as HR, minute ventilation, BT, etc. were chosen from the same period of measured REE.

a, age; ABW, adjusted body weight; ACCP, American College of Chest Physicians; B, burn (yes = 1, no = 0); BMI, body mass index; BSA, body surface area; BT, body temperature; h, height; HBE, Harris–Benedict equation; HR, heart rate; IBW, ideal body weight; MSJ, Mifflin–St. Jeor; RR, respiratory rate; REE, resting energy expenditure; S, sex (male = 1, female = 0); T, trauma (yes = 1, no = 0); Tmax, maximal body temperature; Ve, minute ventilation; VT, tidal volume; w, weight.

### Eligibility criteria

This study included data from ventilated Thai patients who underwent REE measurement by IC, using an E-COVX module integrated in Engstrom ventilators, GE healthcare. The E-COVX calculates REE by measuring oxygen consumption (VO_2_) and carbon dioxide production (VCO_2_). The VO_2_, VCO_2_, and REE values were continuously measured and calculated throughout the day. The average REE value was recorded once VO_2_ and VCO_2_ were first steady (within a ±5% coefficient of variation over a 20 min period). Additionally, the suction and ventilator setting adjustment procedures were not performed within a 2 h period prior to collecting REE data. To obtain precise measured values, we excluded data from patients with the following conditions: (1) hemodynamic instability (systolic blood pressure <90 mmHg or a decrease in systolic blood pressure >40 mmHg or mean arterial pressure <65 mmHg), (2) respiratory instability or variable respiratory patterns (>20% fluctuation of respiratory rate (RR) or respiratory rate >35/min), (3) high level of ventilator setting: FiO_2_ >60% or positive end expiratory pressure (PEEP) >12 cmH_2_O, (4) variations in carbon dioxide pool, such as patients receiving continuous hemodialysis or bicarbonate infusion, and (5) an air-leakage problem, including leaks around ventilator circuits and leaks in chest drainage system [14].

### Statistical analyses

The accuracy of predictive equations was analyzed based on the following norm: A predictive equation was deemed to be accurate when the calculated values arising pursuant to its use fell within ±10% of the measured REE. Results were expressed as numbers and percentages of patients with precise, overestimated, and underestimated REEs, which were calculated by each equation. The discrepancy between estimated and measured values was expressed as a mean percentage error (bias%). Spearman correlation coefficient and the Bland–Altman method were used to test the association and agreement between these compared values [15]. The intraclass correlation coefficient (ICC) (2-way mixed effect) was also used to assess inter-rater agreement. We also compared the accuracy of equations in obese and non-obese patients. Additionally, we used the STARD checklist when writing our report [16].

## Results

**Figure 1.**
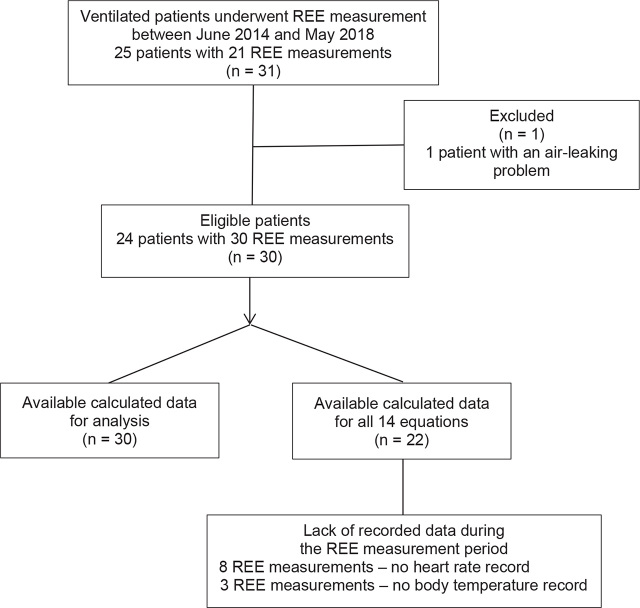
Flow of participants.

Thirty-one measured REEs were obtained from 25 ventilated Thai patients. One patient was excluded due to air leakage during IC measurement (**Figure 1**). The eligible patients had a median age of 64.5 [43.5, 75.5] years, a median height of 160 [151, 167] cm, and a median body mass index (BMI) of 22.95 [18, 34.1] kg/m^2^. The baseline characteristics of 24 eligible patients are summarized in **Table 2**.

**Table 2.** Baseline characteristics of the patients

**Baseline characteristics**	
Sex n (%)	
Male	12 (50)
Age	
Median (years)	64.5 [43.50, 75.50]
Height	
Median (cm)	160 [151.0, 167.0]
Median (kg)	
Median (cm)	62.5 [47.75, 85.50]
BMI n (%)	27 (100)
Underweight (<18.5 kg/m^2^)	6 (25)
Normal (18.5–22.9 kg/m^2^)	7 (29.2)
Overweight (23–24.9 kg/m^2^)	0 (0)
Obese 1 (25–29.9 kg/m^2^)	1 (4.2)
Obese 2 (>30 kg/m^2^)	10 (41.7)
BSA (Dubois) median (m^2^)	1.7 [1.46, 1.86]
Cause of respiratory failure n (%)	
Pneumonia	4 (16.7)
Post ARDS	3 (12.5)
COPD	2 (8.3)
Chronic lung disease	1 (4.2)
Sepsis	2 (8.3)
Cardiac arrest	1 (4.2)
Heart failure	2 (8.3)
Tetanus	1 (4.2)
Others	8 (33.3)
Ventilator mode n (%)	
Volume control	3 (10)
Pressure control	6 (20)
Pressure support	20 (66.7)
Proportional assist ventilation	1 (3.3)
Measured REE median (kcal/d)	
Total	1553.0 [1126.75, 2065.75]
Underweight (<18.5 kg/m^2^)	1044.0 [884.50, 1591.50]
Normal (18.5–22.9 kg/m^2^)	1545.0 [1125.75, 1981.00]
Overweight (23–24.9 kg/m^2^)	–
Obese 1 (25–29.9 kg/m^2^)	1475.0 [1475.00, 1475.00]
Obese 2 (>30 kg/m^2^)	2043.5 [1525.25, 2329.50]
Average measured calories per actual BW (kcal/kg)	23.5
Average measured calories per BW (kcal/kg)	27
Use actual BW if BMI <30 kg/m^2^	
Use adjusted BW if BMI ≥ 30 kg/m^2^	
Average measured calories per IBW (kcal/kg)	29.5
REE calculated by predictive equations median (kcal/d)	
ACCP	1255.0 [1050.00, 1423.00]
HBE	1180.5 [1078.89, 1518.95]
1.2×HBE	1424.3 [1292.80, 1752.98]
1.5×HBE	1780.4 [1615.99, 2191.23]
MSJ	1250.0 [1012.25, 1522.50]
Ireton-Jones 1992	1688.0 [1585.00, 2042.25]
Ireton-Jones 2002	1479.5 [1373.25, 1957.00]
Penn State 2003 (HBE)	1523.5 [1270.24, 1734.94]
Penn State 2003 (MSJ)	1516.5 [1182.04, 1905.00]
Penn State 2010	1542.4 [1310.89, 1846.61]
Swinamer 1990	1740.0 [1502.28, 2115.50]
Faisy	1840.4 [1576.60, 2155.20]
Brandi 1999	1547.3 [1302.86, 1794.43]
Equation commonly used in critical illness (25 kcal/kg)	1562.5 [1225.00, 2400.00]

ACCP, American College of Chest Physicians equation; ARDS, acute respiratory distress syndrome; BMI, body mass index; BSA, body surface area; BW, body weight; COPD, chronic obstructive pulmonary disease; HBE, Harris–Benedict equation; IBW, ideal body weight; MSJ, Mifflin–St. Jeor; REE, resting energy expenditure.

The patients had a median measured REE of 1553 [1126.75, 2065.75] kcal/d and an average measured REE of 23.5 kcal/kg actual body weight/d or 29.5 kcal/kg ideal body weight (IBW)/d. The median estimated REE values, calculated by 14 equations, ranged from 1180.5 kcal/d to 1840.4 kcal/d. We compared the accuracies of 14 equations (**Table 3**). The accuracies of these equations varied from 6.7% to 48.1%, with the mean differences between the calculated and measured REEs of −320 kcal/d to 440 kcal/d and the mean bias percentages ranging from −13% to 31.8%. The total numbers analyzed in each equation differed noticeably. Some parameters used to calculate REE in some equations might not have been recorded, and some periods might not have been incorporated in the estimations for some equations. To minimize these confounders, we performed sensitivity analysis, where all available data were used (**Table 4**). In the sensitivity analysis, the accuracy of the equations varied from 9.1% to 40.9%, with the mean differences between the calculated and measured REEs of −272.4 kcal/d to 353.5 kcal/d and the mean bias percentages ranging from −10.2% to 32.4% (**Table 4**). Notably, the results of the sensitivity analysis and the total results were similar. Penn State 2010 remained the most precise equation, while Ireton-Jones 1992 remained the least accurate. The ACCP, Harris–Benedict equation (HBE), Mifflin–St. Jeor (MSJ), and Penn State 2003 (HBE) also tended to underestimate calorie requirements. Besides, the 1.2×HBE, 1.5×HBE, Ireton-Jones 1992 and 2002, Penn State 2003 (MSJ), Penn State 2010, Swinamer 1990, Faisy, Brandi 1999, and 25 kcal/kg equations tended to overestimate REEs.

**Table 3.** Accuracy of predictive equations

**Equations**	**n**	**Accurate prediction (%)**	**Under estimation (%)**	**Over estimation (%)**	**Bias (%)**	**Difference** **(Predictive value – measured value) (kcal/d)**		
						**Mean**	**Minimum**	**Maximum**
ACCP	30	23.3	60.0	16.7	−13.0	−320	−1257	617
HBE	30	30.0	60.0	10.0	−4.0	−99	−1063	930
1.2×HBE	30	31.8	21.3	40.9	3.2	−49	−742	631
1.5×HBE	30	18.2	9.1	72.7	29.0	322	−476	979
MSJ	30	33.3	50.0	16.7	−10.8	−200	−1172	919
Ireton-Jones 1992	30	6.7	23.3	70.0	22.2	233	−812	923
Ireton-Jones 2002	30	26.7	23.3	50.0	8.0	43	−1018	665
Penn State 2003 (HBE)	24	25.0	50.0	25.0	1.6	−88	−740	837
Penn State 2003 (MSJ)	27	37.0	22.2	40.7	9.6	100	−502	1056
Penn State 2010	27	48.1	14.8	37.0	11.6	98	−472	1089
Swinamer 1990	27	18.5	11.1	70.4	25.9	295	−336	1191
Faisy equation	27	25.9	0.0	74.1	31.8	366	−176	1397
Brandi 1999	22	22.7	27.3	50.0	13.6	74	−549	1115
25 kcal/kg	30	30.0	20.0	50.0	25.2	440	−933	4030

ACCP, American College of Chest Physicians equation; Accurate prediction%, percentage of patients who had predictive values within 90% and 110% of measured values; Bias%, mean percentage error between predictive equations and measured value; HBE, Harris–Benedict equation; Mean bias, difference between predictive and measured values; MSJ, Mifflin–St. Jeor; Overestimation%, percentage of patients who had predictive values of more than 110% of measured values; Underestimation%, percentage of patients who had predictive values of less than 90% of measured values.

**Table 4.** Sensitivity analysis for the accuracy of predictive equations

**Equations (n = 22)**	**Accurate prediction (%)**	**Under estimation (%)**	**Over estimation (%)**	**Bias (%)**	**Difference** **(Predictive value – measured value) (kcal/d)**		
					**Mean**	**Minimum**	**Maximum**
ACCP	22.7	54.5	22.7	−10.2	−272.4	−1091.0	617.0
HBE	27.3	59.1	13.6	14.02	296.7	−987.1	399.7
1.2×HBE	31.8	27.3	40.9	3.18	−49.1	−742.1	631.3
1.5×HBE	18.2	9.1	72.7	29.0	322.3	−475.7	978.6
MSJ	36.4	50.0	13.6	−9.3	−183.3	−773.0	490.8
Ireton-Jones 1992	9.1	18.2	72.7	25.0	275.5	−421.0	923.0
Ireton-Jones 2002	22.7	27.3	50.0	8.8	53.4	−662.0	665.0
Penn State 2003 (HBE)	22.7	50.0	27.3	2.6	−76.1	−739.9	837.1
Penn State 2003 (MSJ)	27.3	27.3	45.5	9.6	73.0	−501.7	944.4
Penn State 2010	40.9	18.2	40.9	12.1	82.6	−471.9	1088.6
Swinamer 1990	18.2	13.6	68.2	25.9	276.6	−335.2	1190.8
Faisy equation	27.3	0.0	72.7	32.4	353.5	−176.0	1397.2
Brandi 1999	23.5	29.4	47.1	14.1	61.4	−548.6	1115.4
25 kcal/kg	31.8	18.2	50.0	20.6	318.5	−933.0	1867.0

ACCP, American College of Chest Physicians equation; Accurate prediction%, percentage of patients who had predictive values within 90% and 110% of measured values; Bias%, mean percentage error between predictive equations and measured value; HBE, Harris–Benedict equation; Mean bias, difference between predictive and measured values; MSJ, Mifflin–St. Jeor; Overestimation%, percentage of patients who had predictive values of more than 110% of measured values; Underestimation%, percentage of patients who had predictive values of less than 90% of measured values.

**Figure 2.**
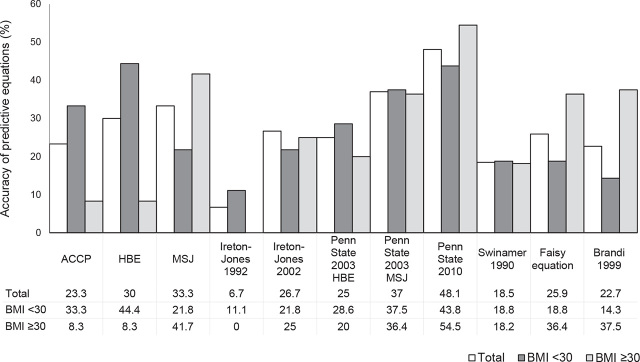
Accuracy of predictive equations for estimating REE in obese and non-obese patients. REE, resting energy expenditure.

In subgroup analysis, the Penn State 2010 equation provided the most accurate estimation of calorie need in obese patients (BMI ≥ 30 kg/m^2^), while the HBE provided the most reliable prediction of REE in non-obese patients (**Figure 2**).

**Figure 3.**
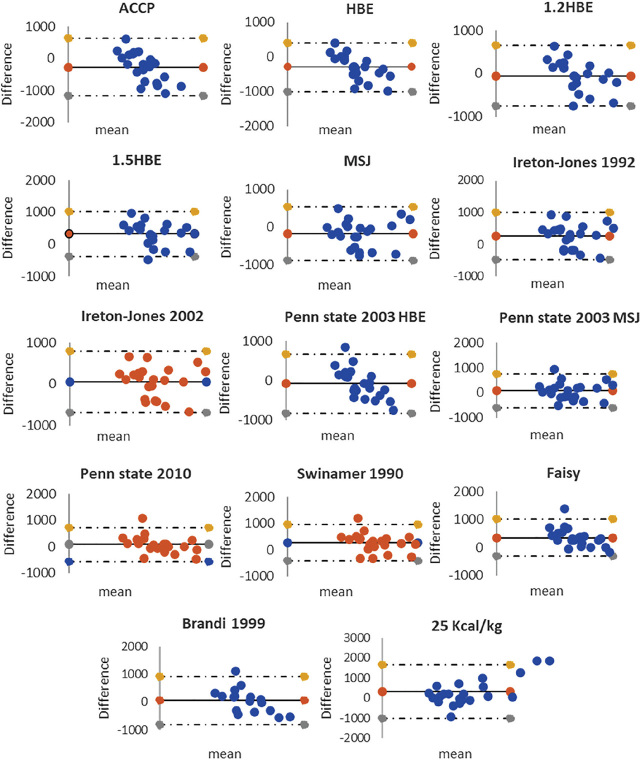
Bland–Altman plots of differences between predictive REE calculated by 14 equations and measured REE (predictive REE – measured REE) in 24 mechanically ventilated Thai patients. The solid lines at the middle part of each picture represent mean differences of compared values and the upper and lower solid lines represent upper and lower limits of agreement (LOA) (±1.96 SD from mean). The points on these plots non-uniformly and widely scatter around mean differences, indicating poor agreement and bias. REE, resting energy expenditure; SD, standard deviation.

Accordingly, on Bland–Altman plots (**Figure 3**), there was a poor agreement between the calculated and measured values. Contrastingly, the calculated values had moderately to strongly positive correlations with measured values (*r* = 0.445–0.778, **Table 5**). Additionally, in the ICC analysis, 1.2×HBE, MSJ, Ireton-Jones 2002, Penn State 2003 (MSJ), and Penn State 2010 had good reliability, while HBE, 1.5×HBE, Ireton-Jones 1992, Penn State 2003 (HBE), Swinamer 1990, Faisy equation, Brandi 1999, and the 25 kcal/kg actual body weight had moderate reliability. Only ACCP had poor reliability (**Table 6**).

**Table 5.** Correlation and agreement between measured REE and predictive REE

**Equations**	**Correlation Spearman rho**	**Bland–Altman**			
		**Mean bias**	**SD**	**Lower LOA Mean – 1.96 (SD)**	**Upper LOA Mean + 1.96 (SD)**
ACCP	0.445	−320	457	−1217	576
HBE	0.675	−99	464	−1008	810
1.2×HBE	0.566	−49	355	−744	646
1.5×HBE	0.566	322	358	−379	1023
MSJ	0.736	−200	429	−1041	641
Ireton-Jones 1992	0.624	233	427	−603	1070
Ireton-Jones 2002	0.666	43	402	−745	831
Penn State 2003 (HBE)	0.714	−88	367	−807	631
Penn State 2003 (MSJ)	0.767	100	369	−623	823
Penn State 2010	0.771	98	312	−513	709
Swinamer 1990	0.708	295	335	−363	952
Faisy equation	0.778	366	333	−287	1019
Brandi 1999	0.587	74	394	−698	846
25 kcal/kg	0.675	439	1019	−4377	2457

ACCP, American College of Chest Physicians equation; HBE, Harris–Benedict equation; LOA, limits of agreement; MSJ, Mifflin–St. Jeor; REE, resting energy expenditure; SD, standard deviation.

**Table 6.** The ICC of 14 equations

**Equations (n = 22)**	**ICC**	**95% Confidence interval**		**F test**
		**Lower bound**	**Upper bound**	
ACCP	0.324	−0.338	0.692	0.136
HBE	0.608	−0.023	0.845	0.003
1.2×HBE	0.764	0.430	0.902	<0.001
1.5×HBE	0.686	0.017	0.886	<0.001
MSJ	0.809	0.515	0.923	<0.001
Ireton-Jones 1992	0.723	0.204	0.894	<0.001
Ireton-Jones 2002	0.800	0.519	0.917	<0.001
Penn State 2003 (HBE)	0.672	0.217	0.863	0.007
Penn State 2003 (MSJ)	0.843	0.626	0.934	<0.001
Penn State 2010	0.814	0.561	0.922	<0.001
Swinamer 1990	0.715	0.155	0.893	<0.001
Faisy equation	0.643	−0.123	0.873	<0.001
Brandi 1999	0.608	−0.104	0.859	0.039
25 kcal/kg	0.701	0.295	0.875	0.002

ACCP, American College of Chest Physicians equation; HBE, Harris–Benedict equation; ICC, Intraclass correlation coefficient; MSJ, Mifflin–St. Jeor. The ICC values of less than 0.5, between 0.5 and 0.75, between 0.75 and 0.9, and greater than 0.90 are indicative of poor, moderate, good, and excellent reliability, respectively.

## Discussion

Our study demonstrated that no predictive equations accurately estimated calorie demand in ventilated Thai patients, and none had clinically acceptable accuracy, despite the fact that calculated values had moderately to strongly positive correlations with measured values and moderate to good reliability in the ICC analysis for inter-rater agreement measurement.

However, HBE more reliably predicted REE in under-weight (BMI < 18.5 kg/m^2^) and non-obese (BMI < 30 kg/m^2^) ventilated patients with the accuracies of 66.7% and 44.4%, respectively, while the Penn State 2010 equation more accurately determined energy need in obese patients with the accuracy of 54.5%. Additionally, the 1.2×HBE, 1.5×HBE, Ireton-Jones 1992 and 2002, Penn State 2003 (MSJ), Penn State 2010, Swinamer 1990, Faisy, Brandi 1999, and 25 kcal/kg equations tended to overestimate REE. Contrastingly, ACCP, HBE, MSJ, and Penn State 2003 (HBE) tended to underestimate energy requirements. The results were consistent with those in a systematic literature review [17]. Tatucu-Babet et al. [17] showed the variability in the accuracy of each equation. The Ireton-Jones, Swinamer, Faisy, and Brandi equations frequently overestimated energy need, while the HBE and Penn State equations were likely to underestimate REE.

Accordingly, the discrepancy between measured and predictive REEs could be explained by the following reasons. First, most equations use body weight (BW) as the basis in their estimation of calorie requirement. Practically, it is difficult to obtain the precise actual weight in mechanically ventilated patients. Commonly, excessive total body water from fluid resuscitation or decreased muscular mass confounds the real values. Second, dynamic changes in clinical conditions result in variable metabolic demands, which depend on phases of critical illness. Furthermore, most equations are validated for healthy volunteers, spontaneously breathing patients, and non-Asian populations, who possibly have metabolic differences from Thais. Finally, there are several factors pertaining to the ICU that might be expected to confound measured values, for example, recent adjustment of the ventilator setting before IC measurement [18], or measurement of calories at different points of time or under the prevalence of different feeding conditions, including fasting, intermittent feeding, or continuous feeding.

Although the predictive equations were initially developed in the western populations, these equations did not precisely estimate REE in mechanically ventilated western patients. Similarly, all of the equations had poor predictive precision in mechanically ventilated Thai patients. Therefore, REE should be assessed using IC.

However, there were some limitations in this study. First, feeding conditions at the time of IC measurement were not recorded. Additionally, the REE was measured during a stable condition, which did not represent energy need in an active phase of illness. Furthermore, there was a selective bias resulting from the fact that physicians usually performed IC in patients with extreme BMI or uncertain actual BW. Finally, the study lacked statistical power due to a small sample size.

## Conclusion

The accuracy of the predictive equations was poor, and thus this method of calorie-need estimation was unable to replace IC in the determination of calorie requirement in mechanically ventilated Thai patients.
